# Can differential nutrient extraction explain property variations in a predatory trap?

**DOI:** 10.1098/rsos.140479

**Published:** 2015-03-18

**Authors:** Sean J. Blamires, Dakota Piorkowski, Angela Chuang, Yi-Hsuan Tseng, Søren Toft, I-Min Tso

**Affiliations:** 1 Department of Life Science, Tunghai University, Taichung 40704, Taiwan, Republic of China; 2 Evolution and Ecology Research Centre, School of Biological, Earth and Environmental Sciences, The University of New South Wales, Sydney, New South Wales 2052, Australia; 3 Department of Life Science, National Chung-Hsing University, Taichung 40227, Taiwan, Republic of China; 4 Department of BioScience, Aarhus University, Building 1540, Ny Munkegade 116, Aarhus 8000 C, Denmark

**Keywords:** animal architecture, extended phenotype, mechanical properties, nutrient-specific foraging, orb webs, trap-building predator

## Abstract

Predators exhibit flexible foraging to facilitate taking prey that offer important nutrients. Because trap-building predators have limited control over the prey they encounter, differential nutrient extraction and trap architectural flexibility may be used as a means of prey selection. Here, we tested whether differential nutrient extraction induces flexibility in architecture and stickiness of a spider's web by feeding *Nephila pilipes* live crickets (CC), live flies (FF), dead crickets with the web stimulated by flies (CD) or dead flies with the web stimulated by crickets (FD). Spiders in the CD group consumed less protein per mass of lipid or carbohydrate, and spiders in the FF group consumed less carbohydrates per mass of protein. Spiders from the CD group built stickier webs that used less silk, whereas spiders in the FF group built webs with more radii, greater catching areas and more silk, compared with other treatments. Our results suggest that differential nutrient extraction is a likely explanation for prey-induced spider web architecture and stickiness variations.

## 2. Introduction

Predators face many challenges in their search for and selection of prey. Because the availability of suitable prey may be spatially or temporally unreliable [1,2], it may be imperative for predators to have flexible foraging strategies so they can effectively switch strategies upon assessment of certain cues [3,4]. The most appropriate cues used at any instance may depend on the sensory modalities of the predator and/or specific characteristics of its preferred prey [5,6].

The balance of proteins, carbohydrates and lipids in prey influence the growth, size, age at maturity and reproductive output of predators [7–9]. For example, when the lipid-to-protein ratio ingested by predatory beetles deviates from a measurable optimum it results in a reduction in egg production [9], likewise wolf spiders on diets containing an optimal protein-to-lipid ratio mature faster [10,11]. Thus, the nutritional value of different prey may be a characteristic that predators use to flexibly select prey [10,12]. Stationary predators that build traps, such as spiders, caddisfly and ant lions, encounter a limited range of prey, partly, because the trap's architecture necessitates that certain prey types are captured more effectively than others [4,13,14]. Accordingly, varying architecture and/or the physical properties of the trap is a strategy that some trap-building predators use to meet their nutritional needs [13,15].

Web-building spiders vary the architectural features of their webs when exposed to different prey or prey differing in nutrient quantity [4,13,16]. In orb-webs, the architectural features that vary include: (i) the number of radial threads, (ii) the size of the web capture area, (iii) the length of the sticky spiral threads, and (iv) spacing between the spirals; or mesh height [4,13,15,16]. Additionally, web-building spiders alter the physical properties of their silks when exposed to different prey or prey of different nutrient quantities [16–19]. These variations in web architecture and properties may be presumed to be a product of the spider building webs to most effectively capture the most nutritiously valuable prey in its environment [4,13,20].

Web building by spiders involves the selection of a suitable location using environmental cues and prior experience [21–23]. The spider then makes decisions about web architecture and silk investment based on space availability, various environmental cues and its energetic or nutritional status [13,24,25]. Once these decisions are made, the spider builds a web following stereotyped behaviours [23,26]. Prey handling behaviours include the act of attacking, subduing and wrapping prey. The sequence of these behaviours depends on the species of spider and/or the prey type or size [27,28].

In a recent study [4], we fed giant wood spiders, *Nephila pilipes*, live crickets, live flies, dead crickets with the web stimulated using live flies or dead flies with the web stimulated using live crickets. We found that spiders fed live flies built orb-webs with a larger capture area, used more radial and sticky spiral threads that were less stiff, and propagated vibrations less efficiently. Accordingly, we deduced [4] that *N. pilipes* builds webs to preferentially catch crickets in the presence of either cricket-induced tactile cues or upon encountering crickets. This response is presumably initiated because crickets were larger and contained more protein. There were, nonetheless, some contradictions to this presumption that we could not explain. For instance, *N. pilipes* fed crickets with fly-induced stimuli made stickier webs [4].

Variations in orb web stickiness are usually a direct consequence of changes in axial thread width, and the number, volume or biochemical composition of the glue droplets that coat the sticky spirals [29–32]. We thus hypothesized that the construction of stickier webs by *N. pilipes* fed dead crickets while receiving fly tactile stimuli is a result of the spiders producing threads and glues with different physical or chemical properties as a consequence of extracting nutrients differently from crickets when receiving fly stimuli. We tested this hypothesis here by replicating the feeding procedures of Blamires *et al.* [4] and determining the amount of protein, carbohydrate and lipid extracted from prey fed to individual *N. pilipes* over seven feeding rounds, then measuring web architectures, silk investment, thread stickiness and glue droplet morphology upon completion of the experiment. Our objective was to assess the mechanism behind prey-induced trap (in this case, a spider orb web) architecture and property (in this case, web stickiness) variability by asking the question: can differential extraction of nutrients explain the architectural and spiral stickiness variations in *N. pilipes* webs resulting from feeding on different prey?

## 3. Material and methods

### 3.1 Spider collection and pre-treatment feeding

We collected 60 subadult female *N. pilipes* (15–20 mm in body length) from Taichung County, Taiwan, and returned them to Tunghai University. We measured their body length to ±0.1 mm and mass to ±0.001 g using digital callipers and an electronic balance, respectively, before placing them in 120 *mm*(*width*)×90 *mm*(*height*) transparent circular containers. The containers had perforated wire mesh lids with a 20 mm slit cut into the mesh screen lids using a Stanley knife to facilitate feeding with a 20 μl micropipette. We fed all of the spiders 20 μl of a 30% w/v sucrose solution daily over 5 days [31]. We weighed all spiders before and after pre-treatment feeding and any spiders that lost more than 20% of their body weight during the pre-treatment feeding were not used in the ensuing experiment. Following pre-treatment feeding, we placed each spider in individual 900×900×330 mm plywood enclosures with front and back Perspex screens within a greenhouse and left them for 3 days to build orb webs.

### 3.2 Feeding experiment

We used laboratory-reared house flies, *Musca domestica*, and crickets, *Acheta domestica* fed standard media ad libitum. The flies and crickets fed to spiders were randomly selected from laboratory colonies and were killed by lethal exposure to carbon dioxide. We did not ascertain the age or sex of any insects that were fed to spiders. The following experiment replicated the feeding regimes of Blamires *et al.* [4] and Tso *et al.* [16,33] under naturally fluctuating temperature, humidity and daylight cycles.

Upon building a web, each spider was randomly assigned to one of four treatment groups (abbreviations following Blamires *et al.* [4]): FF (fed live flies), CC (fed live crickets), FD (fed dead flies but their webs were stimulated by live crickets) or CD (fed dead crickets but their webs were stimulated by live flies). Two spiders failed to build a web within 3 days of completing pre-treatment feeding so were not used further. Subsequently, the FF and CC treatment groups each contained 15 spiders, and the FD and CD treatment group contained 14 spiders. Spiders assigned to the CC treatment had a live cricket placed randomly on their web. Spiders assigned to the FF treatment had five live flies placed randomly on their web. Spiders assigned the FD treatment had a live cricket placed randomly on their web, which was removed and replaced by five freshly killed flies upon the spider responding to the cricket-induced web stimuli, but before the spider captured the cricket [4]. Spiders assigned to the CD treatment group had five live flies placed on the web, which were removed and replaced with one freshly killed cricket [4,16,18]. The rationale of these treatments was that spiders fed the CC and FF treatments had their prey encountered and tactile stimuli coupled, whereas those fed the CD and FD treatments had their prey encountered uncoupled from the tactile stimuli received [4,18]. Effectively, the CC and CD treatments examined the responses of spiders to cricket encounters, and the FF and FD treatments examined the response of spiders to fly encounters, whereas the CC and FD treatments examined the responses of spiders to cricket stimuli and the FF and CD treatments examined the responses of spiders to fly stimuli.

The day that the first round of feeding commenced was considered day 1, and the first web built by each spider once feeding commenced was considered web 1. Within 12 h of each spider completing feeding (indicated by the spider showing no more interest in the remaining prey), we removed the remains of the prey from the web or base of the enclosure and destroyed the web. All spiders were monitored daily for a new web, after which it received another round of feeding. We iterated the experiment until its termination on day 21. Spiders that failed to build at least seven webs (one each from the FD and CD treatments) or died (one from the FF treatment) over 21 days were not included in the subsequent analyses.

### 3.3 Nutrient analyses

Approximately 30 crickets and 150 flies were reared to adulthood and sacrificed by carbon dioxide exposure and freezing. These, and the prey remains removed from webs or enclosures post-feeding, were used for the following nutrient analyses to enable us to precisely ascertain the nutrients extracted from prey across seven feeding rounds.

Whole prey and unconsumed prey remains were dried at 60°C for 48 h in a drying cabinet (DO45; Deng Yng Instruments, Tainan, Taiwan) and weighed to the nearest 0.1 mg using an electronic balance. The mass consumed for each prey item was calculated as the dry mass of the prey remains subtracted from the mean dry mass of whole prey. Upon weighing, all of the samples were placed in 2 ml Sarstedt Microtubes, frozen at −20°C and transported to Aarhus, Denmark.

We extracted lipids from all samples by soaking them three times in petroleum ether, upon which they were vacuum dried. Particularly large samples and whole prey samples were soaked five times. The quantity of lipids removed was calculated as the difference in the mass of the samples before and after soaking. Because we wanted to determine the protein content of many small (less than 1 mg) samples, a direct measure of protein could not be made, so we measured nitrogen content by combustion of the weighed vacuum-dried samples in an NC elemental analyser (NA2000, Carlo Erba, Rodano, Italy) and calculated the crude protein content by multiplying nitrogen content by 6.25 [34]; a method used in previous studies investigating predator nutrition [8,10–12,35,36]. We concede that other forms of insect nitrogen, such as chitin and nucleic acids, may have contributed towards crude protein determination. We, however, did not expect these forms of nitrogen to be differentially extracted by spiders across feeding treatments, so were unlikely to affect our analyses. Total carbohydrates were determined using a modified Anthrone test [37].

### 3.4 Web architectural measurements

We rendered the webs visible after the pre-treatment feeding and after 21 days of experimentation (the measurements made at this time are hereon referred to as the ‘post-treatment measurements’) by gently spraying them with tap water. For each web, we counted the number of radii and sticky spirals along the four cardinal directions (up, down, left and right), and measured (±0.1 mm) the upper, lower, left and right hub and web radii.

These variables were used to calculate:

1. catching area [38]:
  $$\left[\frac{\pi {\left(r_{\mathrm{au}}\right)}^{2}}{2}-\frac{\pi {\left(Hr_{u}\right)}^{2}}{2}\right]+\left[\frac{\pi {\left(r_{\mathrm{al}}\right)}^{2}}{2}-\frac{\pi {\left(Hr_{l}\right)}^{2}}{2}\right],$$
  $$\begin{array}{r} \text{where}\;r_{\mathrm{au}}=\frac{r_{u}+(d_{h}/2)}{2},\\ r_{\mathrm{al}}=\frac{r_{l}+(d_{h}/2)}{2}, \end{array}$$
  and *r*_u_ is the radius of the upper portion of the web, *r*_l_ is the radius of lower portion of web, *d*_h_ is the width of web, *Hr*_u_ is the radius of upper portion of hub and *Hr*_l_ is the radius of lower portion of hub;
2. total silk length [16]:
  $$\text{total silk length}=\pi ({\bar{X}}_{\mathrm{Rweb}}+{\bar{X}}_{\mathrm{Rhub}}){\bar{X}}_{\mathrm{spiral}}+({\bar{X}}_{\mathrm{Rweb}}+{\bar{X}}_{\mathrm{Rhub}}){\bar{X}}_{\mathrm{radii}},$$
  where ${\bar{X}}_{\mathrm{Rweb}}$ is the average radius of web, ${\bar{X}}_{\mathrm{Rhub}}$ is the average radius of hub, ${\bar{X}}_{\mathrm{spiral}}$ is the average number of sticky spirals and ${\bar{X}}_{\mathrm{radii}}$ is the average number of radii;
3. total spiral length, using the formula [16]
  $$\text{total spiral length}=\pi ({\bar{X}}_{\mathrm{Rweb}}+{\bar{X}}_{\mathrm{Rhub}}){\bar{X}}_{\mathrm{spiral}},\;\mathrm{and}$$
4. mesh height [15]:
  $$\text{mesh height}=\frac{\left(r_{u}+r_{l})-(Hr_{u}+Hr_{l}\right)}{S_{u}+S_{l}-2},$$
  where *S*_u_ is the number of spirals in the upper half of the web and *S*_l_ is the number of spirals in the lower half of the web.

### 3.5 Spiral stickiness and droplet measurements

We collected eight sticky spiral threads per web on 76×26 *mm* cardboard cards with an open-ended 14.3 mm hole punched at one end [39]. We lightly touched the tips of the card openings on a spiral thread and held it still for approximately 2 s to allow the thread to adhere to the card border. The treads spanning the card opening were then freed from the rest of the web using a stick of burning incense. The threads were reinforced at the attachment sites using a drop of Elmer's glue. Because only spiral threads with more than 14.3 mm spacing between radii could be collected, all of the spirals were taken from the outermost region of the webs.

We used four spiral thread samples per web for the following stickiness measurements. We placed the top of the cards (i.e. so the open end of the card faced downward) in the uppermost grips of a Nano Bionix tensile tester (MTS Systems Corporation, Oakridge, TN), and a 6×2 *mm* stainless steel stage was mounted securely in the lowermost grips. We then lowered the card at 0.01 mms^−1^ until the thread touched the stage. The specimen was held in position for 60 s, allowing the thread to adhere to the stage, before being pulled up at 0.1 mms^−1^ until the thread detached from the stage. The force (μN) required to pull the thread off the stage was measured as a proxy of stickiness of the thread [31,40] using the program TestWorks v. 4.0 (MTS Systems Corporation). We repeated this procedure 10 times using a different part of the stage each time, obtaining an average value per thread.

The remaining four spiral thread samples taken from each web were used to measure the morphology of the glue droplets. The cards containing spiral threads were gently placed onto parallel matchsticks that were 20 mm apart on a microscope slide, ensuring that the threads and their droplets had no contact with any surface that could distort their shape. We viewed and photographed the spirals under 100× and 1000× magnification using a light microscope (Leica DM 750, Leica Microsystems, Wetzlar, Germany) connected to an SLR digital camera (EOS 600D, Canon, Tokyo, Japan). We calculated the number of glue droplets per 0.5 mm of thread and measured the length and width of 10 randomly selected droplets from the photographs using VIS Plus (Liion Opto-Electronics Technology, Taichung, Taiwan). We removed the dowels, placed the samples back on the slides and re-photographed the threads with the droplets flattened to render the underlying thread visible. We then measured the width of the underlying thread using the program ImageJ (NIH, Bethesda, MD).

We used the above measurements to determine the mean droplet volume (DV) using the formula [29,41]

$$\mathrm{DV}=\frac{2\pi {\left(h\right)}^{2}b}{15},$$

where *h* is half the width of the droplet and *b* is half the length of the droplet. We then calculated the average spacing between droplets (DS) and the droplet volume along 0.5 mm of thread (DV/0.5 mm) [29,30]. We calculated the surface-area-to-volume ratio of the droplets by first calculating droplet surface area (DSA) using the formula [42]

$$\mathrm{DSA}=\frac{4\pi hb}{3}.$$

The droplet surface-area-to-volume ratio (DSA : DV) was then calculated as the DSA divided by DV. All measurements were done as soon as possible after collection, and the treatments were sampled in a random order. Given orb web glue droplets retain their shape and stickiness for several months when stored under laboratory conditions [43], the time taken after web building to perform these measurements (approx. 3–6 days) had negligible effects on any of the between treatment variations.

### 3.6 Analyses

Because our experiment was staged by design, i.e. after each of seven rounds of feeding, we measured the nutrients extracted by the spiders and on day 21 we measured web architecture and stickiness, our analyses were also staged as follows.

First, we compared the mass of prey consumed and the amount of proteins, carbohydrates and lipids extracted from prey across the FF, CC, FD and CD treatments by a repeated measures (each of seven feeding rounds) multivariate analysis of variance (rmMANOVA) and a Fisher's least significant difference *post hoc* analysis. We performed Kolmogorov–Smirnov tests to assess the normality of the data prior to all analyses. We square root- or log-transformed data that failed the tests (*p*<0.05). Because predators will attempt to balance their protein : lipids and/or protein : carbohydrate intake to optimize their fitness [9–12,44], we assessed whether the accumulated ratio of protein : lipid consumed and/or protein : carbohydrate consumed varied across treatments by plotting mean crude protein consumed versus mean lipids consumed, and mean crude protein consumed versus mean carbohydrates consumed for each treatment, accumulated over the seven feeding rounds. We used the data points generated to construct rails through nutrient space [44] for each treatment. The rails were approximations of accumulated protein consumed versus lipids consumed, and accumulated protein consumed versus carbohydrates consumed and were constructed specifically to compare treatments to ascertain whether differential nutrient extraction was likely for any of the treatments.

Second, we used a series of repeated (pre-treatment and post-treatment) measures MANOVAs to determine whether web architecture (i.e. number of radii, catching area, silk length, sticky spiral length and mesh height) or sticky spiral properties (stickiness, thread diameter, DV, DV/0.5 mm and DSA : DV) differed between the FF, CC, FD and CD treatments. Treatment was the independent variable and had four levels: FF, CC, FD and CD. Mauchly's tests were used to test for sphericity (all *p*>0.05). Because variances were heterogeneous for the pre-treatment and post-treatment data (Levene's tests, *p*<0.05), all data were either square root- or log-transformed prior to analysis. Where transformations failed to produce homogeneity, we used non-parametric procedures. We performed Fisher's least-significant difference *post hoc* analyses to ascertain the variables that differed when an rmMANOVA indicated significant differences.

We interpreted any instance of differential nutrient extraction coinciding with web architecture or spiral property variations across treatments as indicative of differential extraction being a probable cause of the web architecture or spiral property variations.

## 4. Results

Body mass did not differ significantly between spiders assigned to the CC, FF, CD and FD treatments after the pre-treatment (Kruskal–Wallis *statistic*=1.617; *p*=0.655), nor did they differ after completion of the experiment (Kruskal–Wallis *statistic*=2.951; *p*=0.399). Thus, we were confident that changes in spider mass did not influence the outcomes of our experiment and that feeding the spiders either one cricket or five flies did not affect the results.

### 4.1 Nutrient extraction

**Figure 1.**
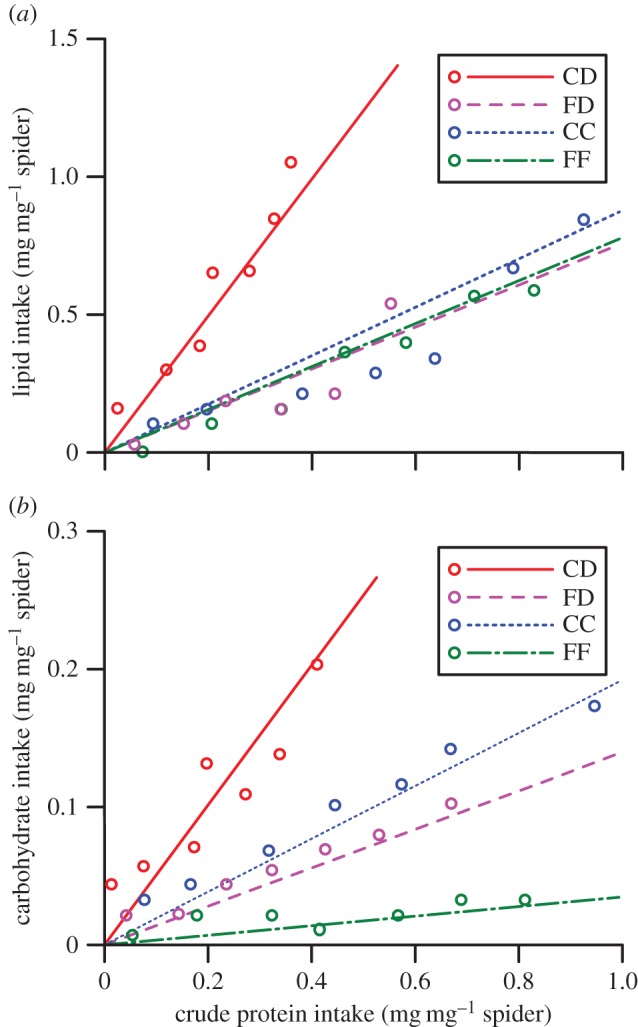
Nutritional rails show the ratio of (*a*) protein : lipid and (*b*) protein : carbohydrates extracted by *Nephila pilipes* fed live crickets (CC), live flies (FF), dead crickets with the web stimulated by live flies (CD) or dead flies with the web stimulated by live crickets (FD) over seven rounds of feeding. Points represent mean values for each round of feeding (1–7).

There was a significant difference in the nutrients extracted by *N. pilipes* across the four treatments over the seven feeding rounds (electronic supplementary material, table S1). *Post hoc* analyses showed that the masses of protein (*F*_3,36_=10.954; *p*<0.001), lipids (*F*_3,36_=2.997; *p*=0.04) and carbohydrates (*F*_3,36_=9.208; *p*<0.001) consumed by spiders (as a proportion of spider mass) differed between treatments across the seven feeding rounds. Inspection of the nutrient rails indicated that the balance of nutrients taken in by spiders in the CD treatment differed from that of the others, with spiders from this treatment consuming a greater mass of carbohydrates and lipids per mass of protein and less nutrients overall (figure 1). Spiders from the FF treatment took in substantially less carbohydrates per mass of protein than spiders from the other treatments (figure 1*b*). Because the mass of prey consumed did not significantly differ across treatments (mean±s.e. masses consumed: *CC*=0.254±0.131, *FF*=0.176±0.082, *CD*=0.236±0.228, *FD*=0.200±0.156 g; *F*_3,36_=2.645; *p*=0.068), we were confident that the different amounts of protein, lipids and carbohydrates consumed by spiders in the CD treatment and the different amounts of carbohydrates consumed by spiders in the FF treatment was owing to differential nutrient extraction.

### 4.2 Web architecture

**Figure 2.**
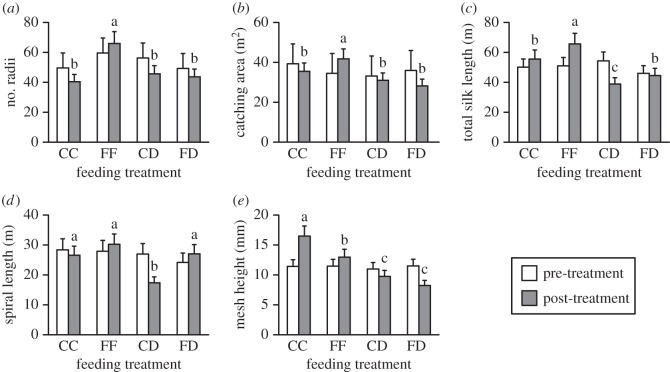
Pre-treatment (empty bars) compared with post-experiment (filled bars) values (mean±s.e.) for: (*a*) number of radii, (*b*) web catching area, (*c*) total silk length, (*d*) spiral length, and (*e*) mesh height for *Nephila pilipes* fed live crickets (CC), live flies (FF), dead crickets with the web stimulated by live flies (CD) or dead flies with the web stimulated by live crickets (FD). Letters show the results of Fisher's least significant difference *post hoc* analyses (*a*>*b*>*c*).

We found a significant difference in the architecture of *N. pilipes* webs across treatments (electronic supplementary material, table S2). *Post hoc* analyses found that the number of radii (*F*_3,36_=2.864; *p*=0.045), catching area (*F*_3,36_=2.941; *p*<0.043), total silk length (*F*_3,36_=5.080; *p*=0.003), total spiral length (*F*_3,36_=3.064; *p*=0.036) and mesh height (*F*_3,36_=4.489; *p*=0.007), differed between treatments. Spiders from the FF treatment built webs with more radii and larger catching areas compared with spiders from the other treatments (figure 2*a*,*b*). Total silk length was lower for webs from spiders in the CD treatment and greater for webs from spiders of the FF treatment compared with the other treatments (figure 2*c*). Spiral length was lower for webs from spiders in the CD treatment compared with all other treatments (figure 2*d*), and mesh height was greater for webs from spiders in the CC treatment compared with all other treatments (figure 2*e*).

### 4.3 Spiral properties

**Figure 3.**
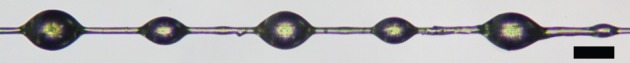
Photograph (scale bar, 20 μm) of a representative sample of *N. pilipes* spiral silk showing that there were two distinct sizes of droplets: large (primary) droplets and small (secondary) droplets.

We observed two distinctly different-sized spiral droplets (figure 3). We therefore followed the terminology of Opell & Hendricks [30] and designated the larger droplets as ‘primary’ droplets and the smaller droplets as ‘secondary’ droplets. We calculated DV and DSA : DV for the primary and secondary droplets separately and included both in our analyses. The number of primary (Kruskal–Wallis *statistic*=3.847; *p*=0.278) and secondary (Kruskal–Wallis *statistic*=6.497; *p*=0.092) droplets did not vary across treatments, so we included both droplet types into a singular measure of DV/0.5 mm [30].

**Figure 4.**
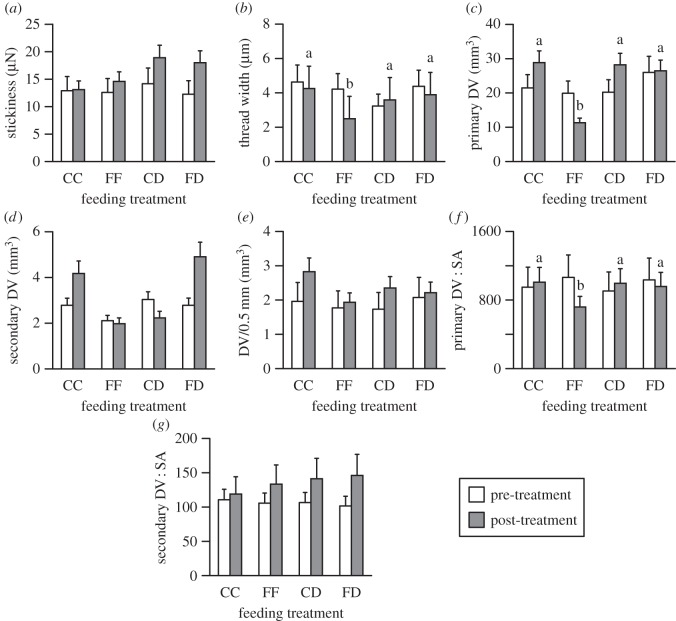
Pre-treatment (empty bars) compared with post-experiment (filled bars) values (*mean*±*s*.*e*.) for: (*a*) spiral stickiness, (*b*) spiral thread width, (*c*) primary droplet volume (DV), (*d*) secondary droplet volume (DV), (*e*) droplet volume per 0.5 mm of spiral thread (DV/0.5 mm), (*f*) primary droplet volume-to-surface area ratio (DV : SA), and (*g*) secondary droplet volume-to-surface area ratio (DV : SA) for *Nephila pilipes* fed live crickets (CC), live flies (FF), dead crickets with the web stimulated by live flies (CD) or dead flies with the web stimulated by live crickets (FD). Letters show the results of Fisher's least significant difference *post hoc* analyses (*a*>*b*>*c*).

We found significant differences in *N. pilipes* sticky spiral thread properties across treatments (electronic supplementary material, table S3). *Post hoc* analyses showed that spiral thread diameter (*F*_3,36_=2.864; *p*=0.045), DV (*F*_3,36_=5.282; *p*<0.001) and DSA : DV (*F*_3,36_=7.583; *p*<0.001), of the primary droplets differed between treatments (figure 4). For each of these properties, the FF treatment differed from the other treatments (figure 4*b*,*c*,*f*). Hence, low prey carbohydrate extraction per mass of protein extracted appeared to associate with changes in glue droplet morphology. Spiral stickiness varied across treatments, albeit to an insignificant extent (*F*_3,36_=1.825; *p*=0.081), with the spirals of the CD treatment group being stickier than those of any of the other treatments (figure 4*a*).

## 5. Discussion

We found the orb web spider *N. pilipes* to consume less protein per mass of lipids or carbohydrates when fed dead crickets with the web stimulated by live flies (the CD treatment) compared with any other feeding treatment. We also found that spiders in the CD treatment had greater sticky spiral length and overall silk length. Spiders fed live flies (the FF treatment) consumed fewer carbohydrates per mass of protein compared with spiders in any of the other feeding treatments. We also found, as did Blamires *et al*. [4], that spiders in the FF treatment built webs with larger capture areas, using more radial threads. Because nutrient extraction and web architecture showed similar patterns of variation across treatments, we considered differential extraction a likely cause of spider web architecture variation under the influence of diet. Nutrient extraction and spiral thread stickiness, on the other hand, did not show, similar patterns of variation across treatments, so it seems unlikely that variations in web stickiness are a consequence of differential nutrient extraction.

A multitude of studies have shown different prey to induce variations in the architecture of spider webs (reviewed by Herberstein & Tso [45]). While nutrients in particular protein are implicated as inducers of orb-web architectural variations [15,46], how nutrients interact with other prey attributes, such as prey size and tactile stimuli, is difficult to discern [4,13]. We found here that spiders in the CD treatment consumed the least amount of protein per mass of lipid or carbohydrate and invested the least silk in their webs. Similarly, spiders in the FF treatment consumed the least carbohydrates and invested more in radial and spiral threads with smaller glue droplets. These results suggest that the balance of protein and non-protein nutrients may influence the amount and allocation of silk invested by spiders into webs.

We found variation in *N. pilipes'* radii investment across treatments. Other studies [4,13,15,46] found protein consumption to correlate with radii investment by orb-web spiders. We, nevertheless, did not find crude protein consumption to be associated with radii investment herein. Rather, the protein to carbohydrate ratio seemed to be a more important indicator of radial silk investment for spiders in the FF treatment. Most other studies either used considerably smaller spiders than *N. pilipes* or starved the spiders of protein [15], neither of which we did here. We surmise, therefore, that a reduction in radii investment might be induced if the spiders have low protein stores or they experience severe protein deprivation. We hypothesize, as did Blamires *et al.* [4], that the variation in *N. pilipes'* radii investment across treatments is explained by the spiders tuning their web's capture performance to accommodate the prey they perceive, using appropriate cues (of which, carbohydrate : protein balance might be important), as being the largest, most predominant or most nutritious, available [20]. We suggest that the ability of alternative web architectures to withstand particular kinetic energy impacts be tested to confirm this hypothesis.

Spiders in the FF treatment extracted the least carbohydrates per mass of protein and had webs with wider spiral threads and larger glue droplets than spiders from the other treatments, suggesting that the volume of silk invested in a spider's webs might depend on the balance of nutrients consumed. Spiral threads comprise the greatest volume and weight of an orb-web [47,48], so changes in spiral thread width and DV will also result in significant changes in silk volume and distribution within the webs from spiders in the different treatments, with likely consequences on the distribution of stresses within the web [48].

The gluey aggregate silk component of orb web spiral threads contains glycoproteins, salts, neurotransmitters and low molecular weight organic compounds that are synthesized at relatively high metabolic costs [31,32,49]. Our finding that spiders that extracted the least amount of protein also invested the least in spiral threads (i.e. spiders from the CD treatment) suggests that investing in spiral threads comes at a reasonably high nutritional cost. Nevertheless, the underlying causal relationship between protein consumption and spiral thread investment in orb webs is unknown, but it may be related to the nutritional cost of synthesizing the various compounds found in spiral glues [49]. We found that the spiral threads of spiders in the FF treatment had different glue droplet morphologies compared with spiders from the other treatments. We predict that the low carbohydrate to protein intake ratio experienced by spiders on the FF treatment might have affected the composition of compounds in the glue droplets and thus affected the ability of the spiral droplets to absorb water from the atmosphere [31,42].

We found that *N. pilipes* from the CD treatment made stickier spiral threads, but to a statistically insignificant extent. Protein deprivation increases spiral stickiness in the orb-web spider *Nephila clavipes* [31]. Nevertheless, unlike Blamires *et al.* [31], we did not completely deprive our spiders of protein. Perhaps spiders from the CD treatment experienced enough protein deprivation to produce only marginally stickier spirals. The type and concentration of salts and glycoproteins in the glue influences the stickiness of orb web spiral threads [29–32]. We thus suggest that future studies seeking to understand the role of nutrients in inducing orb web stickiness should perform additional biochemical assays on the spiral glue.

A number of studies show that cursorial foraging spiders can extract different quantities of proteins, carbohydrates and lipids from different prey [10,12,50]. Our study, to our knowledge, is the first to show a web-building spider extracting different amounts of proteins, carbohydrates and lipids from the same prey when coupled to different tactile stimuli. We did not examine how the spiders differentially extracted nutrients, but encountering different prey and/or the uncoupling of prey with their tactile stimuli may have induced the spiders to slightly alter their capacity for prey identification, or induced alternative handling behaviours, resulting in the consumption of different components of each of the prey [12]. Uncoupling of the prey and tactile stimuli alone, however, did not induce *N. pilipes* in the CD treatment to extract nutrients differently, because the uncoupling of the prey and tactile stimuli in the FD treatment did not similarly result in the extraction of different amounts of nutrients compared with the CC and FF treatments.

Prey handling includes the act of attacking, subduing and wrapping prey in a sequence dependent on the species of spider and/or prey [27,28]. We observed that prey attacks were slightly (albeit unmeasured) slower when the spiders were fed dead crickets with the web stimulated by live flies or dead flies with the web stimulated by live crickets. However, if different attack behaviours alone explained the differential extraction of nutrients, then the nutrients extracted from the CC and FF treatments should be similar, as should those from the CD and FD treatments. As we did not find this pattern, different attack behaviours on the different prey seem to be unlikely. Subduing and wrapping prey by spiders involves the retention, envenomation and manipulation of prey while secreting and drawing the wrapping silks [51,52]. While these behaviours will differ between individual spiders depending on the prey encountered and various conditions during encounter [53], how behavioural variations cause spiders to extract nutrients differently from different prey is not known. If the subduing and wrapping of crickets is costly, but the nutritional reward of consumption is high, feeding on dead crickets may be highly profitable for spiders and could induce their foraging behaviours to change. This variation in foraging profitability might explain the reason why spiders in the CD treatment differentially extracted nutrients and decreased their investment in radial and spiral threads and used stickier glues.

Predators that use traps have unique nutritional needs, including the need to invest in materials to build the trap [13–15]. Our study implies that the balance of nutrients consumed is associated with the material investment in traps, which may affect trap architecture and performance, providing negative feedback. This might explain the rarity of trap use among predators. Furthermore, it suggests that changes in the nutritional environment may force some spiders to change their silk investment and/or foraging strategies and could explain the perpetual changes in spider web forms over evolutionary time [54].

## Supplementary Material

Electronic supplementary material tables s1-s3

## Data accessibility

Datasets pertaining to this study are deposited in Dryad (http://www.datadryad.org). (doi:10.5061/dryad.t01q1).
