# Supplementary material for: Can differential nutrient extraction explain property variations in a predatory trap?
Source: R Soc Open Sci. 2015 Mar 18;2(3):140479. doi: 10.1098/rsos.140479 (PMC4448829; doi:10.1098/rsos.140479)
Supplement: Electronic supplementary material tables s1-s3 [file rsos140479supp1.docx]

**Table S1** Results of repeated measures (seven feeding rounds) multivariate analyses of variance (rmMANOVA) of the nutrients extracted by *Nephila pilipes* across the four feeding treatments: FF (live flies), CC (live crickets), FD (dead flies but their webs were stimulated by live crickets) and CD (dead crickets but their webs were stimulated by live flies). The multiple dependent variables in the analysis are: proteins, lipids and carbohydrates extracted.

| source | d.f. | Wilk’s λ | *p* |
| --- | --- | --- | --- |
| feeding round | 3,26 | 0.223 | <0.001 |
| treatment | 3,26 | 0.231 | 0.001 |
| feeding round x treatment | 9,82 | 0.168 | <0.001 |

**Table S2** Results of repeated measures (pre-treatment and post-treatment) multivariate analyses of variance (rmMANOVA) of architectural properties of *Nephila pilipes* webs across the four feeding treatments: FF (live flies), CC (live crickets), FD (dead flies but their webs were stimulated by live crickets) and CD (dead crickets but their webs were stimulated by live flies). The multiple dependent variables in the analysis are: catching area, silk length, spiral length and mesh height.

| source | d.f. | Wilk’s λ | *p* |
| --- | --- | --- | --- |
| pre-treatment | 3,36 | 0.722 | 0.001 |
| treatment | 3,36 | 0.858 | 0.595 |
| pre-treatment x treatment | 9,109 | 0.085 | <0.001 |

**Table S3** Results of repeated measures (pre-treatment and post-treatment) multivariate analyses of variance (rmMANOVA) of *Nephila pilipes*’ sticky spiral droplet properties across the four feeding treatments: FF (live flies), CC (live crickets), FD (dead flies but their webs were stimulated by live crickets) and CD (dead crickets but their webs were stimulated by live flies). The multiple dependent variables in the analysis are: stickiness, axial thread diameters, droplet volume, number of droplets per 0.5 mm of thread, and droplet surface area to volume ratio.

| source | d.f. | Wilk’s *λ* | *p* |
| --- | --- | --- | --- |
| pre-treatment | 3,36 | 0.837 | 0.001 |
| treatment | 3,36 | 0.618 | <0.001 |
| pre-treatment x treatment | 9,109 | 0.258 | <0.001 |
